# Modeling and *in vivo* experimental validation of 1,064 nm laser interstitial thermal therapy on brain tissue

**DOI:** 10.3389/fneur.2023.1237394

**Published:** 2023-10-06

**Authors:** Peng Cao, Dingsheng Shi, Ding Li, Zhoule Zhu, Junming Zhu, Jianmin Zhang, Ruiliang Bai

**Affiliations:** ^1^Department of Neurosurgery, Zhejiang University School of Medicine Second Affiliated Hospital, Hangzhou, Zhejiang Province, China; ^2^Research and Development Department, Hangzhou GenLight MedTech Co., Ltd., Hangzhou, Zhejiang Province, China; ^3^Clinical Research Center for Neurological Diseases of Zhejiang Province, Hangzhou, Zhejiang Province, China; ^4^Interdisciplinary Institute of Neuroscience and Technology, School of Medicine, Zhejiang University, Hangzhou, China; ^5^MOE Frontier Science Center for Brain Science and Brain-machine Integration, School of Brain Science and Brain Medicine, Zhejiang University, Hangzhou, China

**Keywords:** laser interstitial thermal therapy, thermal damage, laser ablation, simulation, bioheat transfer

## Abstract

**Introduction:**

Laser interstitial thermal therapy (LITT) at 1064 nm is widely used to treat epilepsy and brain tumors; however, no numerical model exists that can predict the ablation region with careful in vivo validation.

**Methods:**

In this study, we proposed a model with a system of finite element methods simulating heat transfer inside the brain tissue, radiative transfer from the applicator into the brain tissue, and a model for tissue damage.

**Results:**

To speed up the computation for practical applications, we also validated P1-approximation as an efficient and fast method for calculating radiative transfer by comparing it with Monte Carlo simulation. Finally, we validated the proposed numerical model in vivo on six healthy canines and eight human patients with epilepsy and found strong agreement between the predicted temperature profile and ablation area and the magnetic resonance imaging-measured results.

**Discussion:**

Our results demonstrate the feasibility and reliability of the model in predicting the ablation area of 1,064 nm LITT, which is important for presurgical planning when using LITT.

## 1. Introduction

Laser interstitial thermal therapy (LITT) is an emerging, minimally invasive and cytoreductive neurosurgical tool for various central nervous system (CNS) lesions ranging from tumors to epilepsy foci, and non-CNS tumors, such as liver tumors (1–3). Although this technique was first described in 1983 (4), enthusiasm for its use has increased in the last 2 decades owing to advances in magnetic resonance imaging (MRI), which enables using real-time thermometry to monitor and visualize the ablation procedure (3, 5–7). During LITT, the optical fiber catheter is percutaneously introduced into the center of the solid lesion, then the tip of the optical fiber emits light to the surrounding tissue, which induces heating and leads to tumor cell coagulation and necrosis (8, 9). The ablation effect depends on the laser wavelength, duration, and power delivered. In previous literature, the characteristics of 980 nm and 1,064 nm laser wavelengths applied in clinical surgical procedures have been contrasted (6, 10–14). The 980 nm (wavelength) laser is often used and has been well-studied in LITT (15–19); it can produce rapid and localized heating of tissues, thereby causing lesions with distinct borders while using relatively low power (9). The 980 nm laser offers advantages such as concentrated energy, faster heating rates, and clearer boundaries. The 1,064 nm laser has been developed more recently for use in LITT; it offers the benefit of a larger ablation zone because the absorptive properties of water are lower at 1064 nm than at 980 nm, offering greater photon scattering and tissue penetration (8, 20).

Numerical modeling of temperature profiles and ablation boundaries is necessary in presurgical planning and plays a key role in the development and application of LITT (21). In such models, the main factors to be considered are (1) the propagation of radiation and temperature in the targeted tissue, which are typically modeled by systems of partial differential equations; and (2) the tissue damage that models the transition between the natural and coagulated states due to the heat effect. The first numerical model of LITT was presented 3 decades ago and improved researchers’ understanding of the laser effect and enabled optimizing the laser parameters in advance (22). Since then, several numerical models of LITT have been built with different laser parameters and tissue types (1, 23–28). However, experimental validation is also critical to evaluate these numerical models because many tissue properties can significantly affect the heat/radiation propagation. *In vivo* experimental evidence is preferred as tissue properties differ significantly between the *ex vivo* and *in vivo* stages.

The 1,064 nm LITT is widely used to treat brain epilepsy and tumors owing to its advantages in deeper penetration and larger ablation zone (1, 3, 9, 14, 27–30). However, no numerical model exists that simulates its thermal profile and ablation effect in brain tissue with careful *in vivo* validation. Development of this model is highly desired for further use of 1,064 nm LITT in treating brain lesions.

We conducted this study to provide a numerical model with fast computing speed and *in vivo* experimental validation of the 1,064 nm LITT in brain tissue. The proposed numerical model is a system of finite element methods simulating heat transfer inside the brain tissue, radiative transfer from the applicator into the brain tissue, and a model for tissue damage. To quicken the computation for practical applications, we also validated P1-approximation as an efficient and fast method of calculating radiative transfer by comparing it with Monte Carlo simulation. Finally, we validated the proposed numerical model *in vivo* on six healthy canines, six human patients with epilepsy lesions who underwent 1,064 nm LITT and two human patients who underwent 980 nm LITT.

## 2. Methods

### 2.1. Finite element simulation of LITT

#### 2.1.1. Model setup

**Figure 1:**
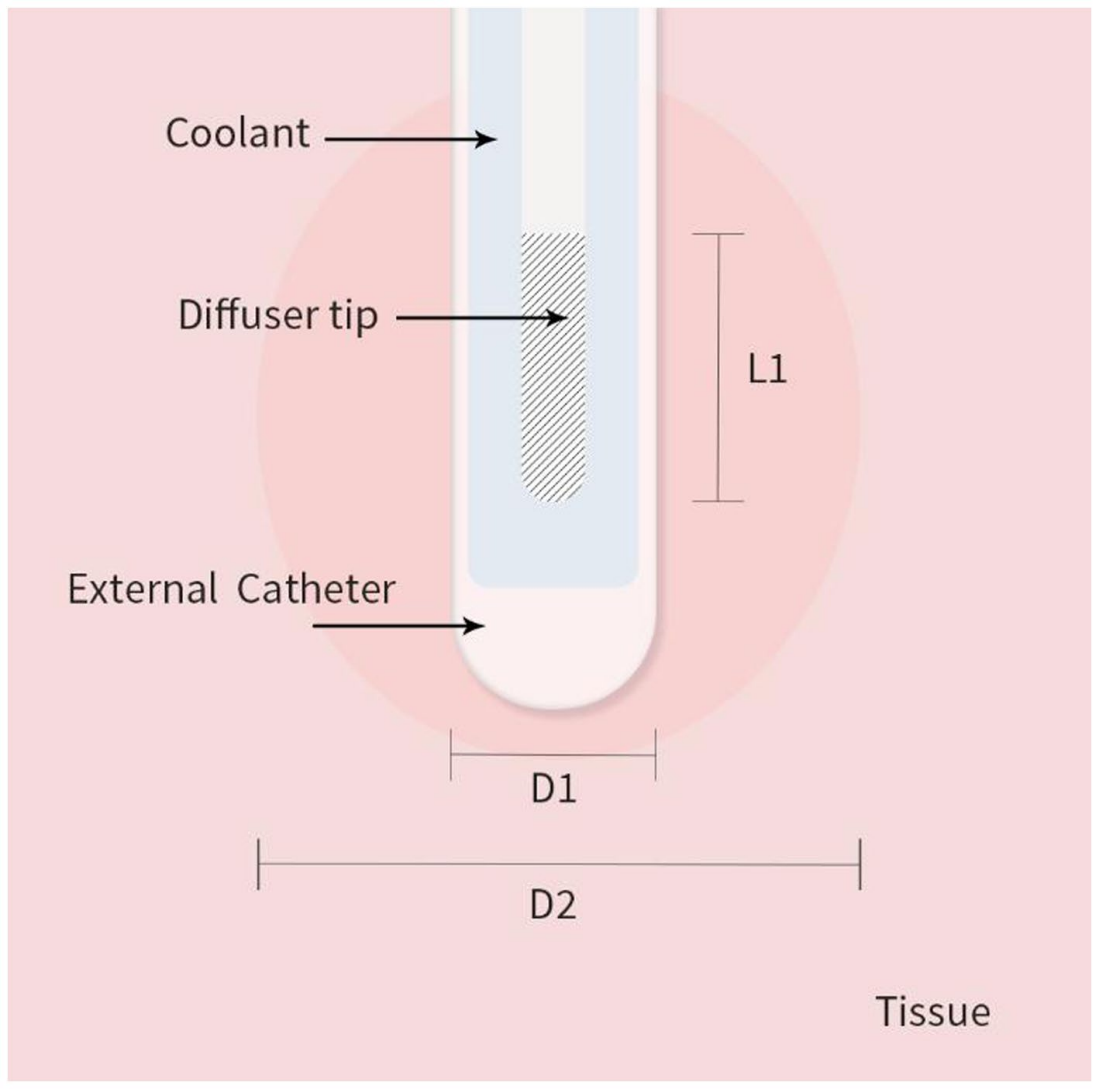
2D axisymmetric brain tissue heating and ablation model with boundary conditions. L1 is the diffusion tip length, D1 is the catheter diameter, and D2 is the final ablation zone diameter induced by LITT.

As shown in Figure 1 we created a two-dimensional (2D) axisymmetric brain tissue model to simulate the spatial distribution of light and temperature during LITT, which involves inserting a diffusing laser applicator into the brain lesion. The model consists of an optical fiber and brain tissue. The optical fiber includes an external catheter, diffusion tip, and coolant, and the brain tissue is assumed to be isotropic and homogeneous. To simplify the model, we set the inner edge of the tube wall as an isothermal surface with a constant temperature of 25°C to simulate the cooling effect of the coolant flow. The diffusion tip columnar luminescence uniformly scatters the laser energy, with two optical diffusing sizes (10 mm and 4 mm for the diffusion tip length) included in the study. We used COMSOL Multiphysics 5.5a (31), a commercial finite element analysis software, to solve partial equations of the heat transfer, radiative transfer, and tissue damage, as described below, which were run on a computer with 32 GB of RAM and eight-core CPU operation at 3.6 GHz. The distance between nodes in the mesh was ~0.2 mm, which provides a good balance between accuracy and computational efficiency in the finite element analysis.

#### 2.1.2. Heat transfer model

To calculate the temperature distribution in brain tissue, we used the Pennes bio-heat transfer model (32), which is based on the principles of energy conservation and heat transfer and describes the temperature distribution within a tissue as a function of time and spatial coordinates. The Pennes bio-heat equation is as follows:


(1) $$\rho c\frac{\partial T}{\partial t}=\nabla .\left(k\nabla T\right)+\rho_{b}c_{b}\omega_{b}\left(T_{b}-T\right)+Q_{m}+Q_{r}$$

where ρ is the tissue density (kg cm^−3^), c is the heat capacity of the brain tissue [J kg^−1^°C^−1^], *k* is the thermal conductivity of the tissue (W m^−1^°C^−1^), T is the tissue temperature (°C), t is the time (s), *ρ*_b_ is the blood density (kg m^−3^), c_b_ is the specific heat capacity of the blood (J kg^−1^°C^−1^], ω_b_ is the blood perfusion rate per unit volume (m^−3^ s^−1^), Tb is the arterial blood temperature, which was 37°C in this study, Q_r_ is internal heat source term due to photon absorption (W m^−3^), Q_m_ is the metabolic heat generation per unit volume, and ∇. is the divergence operator.

The first term on the right-hand side of the equation reflects heat conduction within the tissue; the second term reflects convective heat transfer due to blood perfusion. The third term reflects metabolic heat generation (Q_m_); the fourth term reflects heat generation owing to the absorption of laser photons (Q_r_). However, in LITT, the metabolic activity of the tissue is often ignored (24) because LITT involves heating the tissue to a temperature that causes coagulation; therefore, the heat generation by metabolic activity is too small to be considered.

#### 2.1.3. Radiative transfer model

##### 2.1.3.1. Monte Carlo simulation

Monte Carlo simulation can be used to calculate the fluence rate distribution in tissue by solving the radiative transfer equation, which describes the transport of photons in biological tissue (25, 33). The algorithm allows an arbitrary angular profile of the radiation exiting the applicator tip. This enables simulating different applicator types. Specifically, the light power density was solved by using an open Monte Carlo code (34) in MATLAB 2019a (MathWorks Inc., Natick, MA, United States). The radiative transfer equation can be written as follows:


(2) $$\frac{dI\left(r,s\right)}{ds}=-\left[\begin{array}{l} \mu a\left(r\right)+\\ \mu s\left(r\right) \end{array}\right]I\left(r,s\right)+3\mu s\left(r\right)\int I\left(r^{\prime },s\right)P\left(r^{\prime },r\right)d\Omega$$

where I(r,s) is the fluence rate at position r along direction s, μa(r) is the absorption coefficient, μs(r) is the scattering coefficient, and P(r’,r) is the probability of a photon being scattered from position r’ to r. The integral in the equation represents the scattering of photons from all directions at position r’ to position r.

To solve this equation using Monte Carlo simulation, transport of many photons through the tissue is simulated. At each step, the photon is either absorbed or scattered according to the probability distribution functions of absorption and scattering. The position and direction of the photon are updated after each scattering or absorption event until the photon either escapes the tissue or reaches a predefined depth.

##### 2.1.3.2. P1-approximation

Compared with Monte Carlo simulation, P1-approximation is a simplified and computationally efficient method for solving laser diffusion simulation problems (31, 33). Monte Carlo simulations require tracking many individual photons, which can be computationally expensive. However, P1-approximation simplifies the modeling by assuming a uniform distribution of photons and a single scattering event, reducing the number of variables to be considered.


(3) $$-\nabla \cdot \left(D\nabla \varphi \right)+\mu_{a}\varphi =0$$

The diffusion coefficient D [m] is then defined by


(4) $$D=\frac{1}{3\;\left(\mu a+\mu s\left(1-g\right)\right)}$$

The absorption and scattering coefficients are denoted by μa [m^−1^] and μs [m^−1^], scattering anisotropy in the Henyey-Greenstein phase function (g). They are eigenfunctions of the Laplace operator in spherical coordinates.

#### 2.1.4. Tissue damage model

We implemented the Arrhenius formula, which is widely used to model the tissue damage in thermal ablation therapies (1), including LITT. The formula relates the rate of a chemical reaction to temperature and activation energy (35) and is often used in conjunction with finite element analysis to create a comprehensive simulation model of the LITT process. Simulating the temperature distribution in the tissue during LITT allows using the Arrhenius formula to predict the extent of tissue damage at different locations within the tissue. This damage can be measured using the following equation because it obeys the Arrhenius law (35, 36):


(5) $$\Omega =\overset{\tau }{\underset{0}{\int }}\mathrm{Aexp}\left(\frac{-E_{a}}{RT}\right)dt$$

where A is a pre-exponential factor [s^−1^] for the brain, E is the activation energy [J mol^−1^ K^−1^] for the brain, R is the universal gas constant [J mol^−1^ K^−1^], T is the Kelvin temperature [K^−1^], and t is the total heating time [s^−1^]. The tissue is assumed to be irreversibly damaged when Ω=1, which corresponds to denaturation of 63% of the molecules (23, 35, 37).

#### 2.1.5. Brain tissue properties used in the numerical model

Table 1 shows the optical and thermal parameters of the brain tissue used in this study and the references from which these parameters were taken.

**Table 1:** Optical, thermal, and damage parameters used in the numerical model.

	Parameter	Reference
Optical, brain (~1,064 nm)		
-Absorption coefficient, cm^−1^	0.5	(8)
-Scattering coefficient, cm^−1^	57	(8)
-Anisotropy factor	0.9	(8)
Optical, brain (~980 nm, as the control)		
-Absorption coefficient, cm^−1^	0.9	(8)
-Scattering coefficient, cm^−1^	78	(8)
-Anisotropy factor	0.95	(8)
Brain density, kg/m^3^	1,040	(30)
Heat conductivity [W m^−1^ K^−1^]	0.503	(30)
Heat capacity Cp [J kg^−1^ K^−1^]	3,590	(30)
Damage rate constant A [s^−1^]	3.1e98	(8, 23)
Damage activation energy Ea [J mol^−1^ K^−1^]	6.28e5	(8, 23)
Gas constant R [J mol^−1^ K^−1^]	8.31	(8)
Blood property		
-Density, kg/m^3^	1,050	(30)
-Perfusion rate, 1/s	0.0048	(30)
-Heat capacity Cp [J kg^−1^ K^−1^]	3,640	(30)
Cooling water property		
-Density, kg/m^3^	1,000	(38)
-Heat conductivity [W m^−1^ K^−1^]	0.598	(38)
-Heat capacity Cp [J kg^−1^ K^−1^]	4,137	(38)
Polycarbonate property		
-Density kg/m^3^	1,300	(38)
-Heat conductivity [W m^−1^ K^−1^]	0.341	(38)
-Heat capacity Cp [J kg^−1^ K^−1^]	2,300	(38)

### 2.2. Experimental validation

#### 2.2.1. 1,064 Nm LITT on canine brain tissue

To evaluate the accuracy of the simulation model for the 1,064 nm LITT, we first tested the 1,064 nm LITT (Hangzhou GenLight MedTech Co., Ltd., Hangzhou, China) on six canine brains with real-time MRI temperature monitoring. All animal studies were conducted in accordance with protocols approved by the Zhejiang University Animal Care and Use Committee (Ethics code: ZJU 20210094). Six canines underwent the procedure. A water-cooled diffusing tip laser applicator, laser power levels between 10 and 15 W, and irradiation times of 90–180 s were used.

#### 2.2.2. 1,064 Nm and 980 nm LITT on human patients with focal epilepsy

To further evaluate the accuracy of the LITT simulation model in humans, we tested the 1,064 nm LITT (Hangzhou GenLight MedTech Co., Ltd.) on six patients and 980 nm LITT on two patients with focal epilepsy.

#### 2.2.3. Real-time MRI thermometry (MRT)

To monitor real-time temperature changes during LITT ablation, MRI was performed using the temperature-dependent proton resonance frequency (PRF) method, which has an accuracy of ±2°C in several tissue types (23, 39–41). Phase images were acquired using a fast gradient-spoiled gradient recalled echo (FSPGRE) pulse sequence with acquisition parameters. To convert the phase changes obtained *via* PRF-MRI into temperature changes, a special conversion formula is used:


(6) $$\Delta T=\frac{\Delta \varphi }{2\pi \cdot a\cdot \gamma \cdot B_{0}\cdot T_{E}}$$

where Δφ is the phase change of the FSPGRE signal, ΔT is the temperature change, a is the temperature sensitivity coefficient fixed at −0.010 ppm/°C (42), γ is the gyromagnetic ratio fixed at 42.58 Hz/T, $B_{0}$ is the strength of the main magnet (3.0 T in this study), and $T_{E}$ is the echo time of the FSPGRE sequence.

For the canine MRI experiments, the PRF-MRI was performed on a 3.0 T Skyra MRI (Siemens AG, Erlangen, Germany) with the following imaging parameters: field of view: 210 mm^2^, matrix size: 144 × 144, slice thickness: 5 mm, $T_{E}$: 7 ms, and time per frame: 6 s. A body coil with 18 channels was used. Before turning on the laser, five PRF-MRI frames were acquired as the baseline to calculate the temperature changes in Eq. (6). The baseline temperature of the canine brain tissue was set as 37°C.

For the human MRI experiments, the PRF-MRI was performed on a 3.0 T 750 W scanner (GE Healthcare, Chicago, IL, United States) with the following imaging parameters: field of view: 220 mm^2^, matrix size: 160 × 160, slice thickness: 5 mm, and $T_{E}$: 12 ms. A 6-ch Neuro Flex coil was used. Before turning on the laser, five PRF-MRI frames were acquired as the baseline to calculate the temperature changes in Eq. (6). The baseline temperature of the human brain tissue was set as 37°C.

#### 2.2.4. Other MRIs to determine the ablation area

Immediately after the ablation treatment, T2-weighted images or contrast-enhanced (CE) T1-weighted images (43) were acquired to evaluate the ablation area. In the canine experiments, the T2-weighted images were acquired with a fast spin echo sequence: field of view: 230 mm^2^, matrix size: 512 × 512, slice thickness: 0.6 mm, and effective *T*_E_: 410 ms. For the human experiments, CE T1-weighted MRI was acquired with a gradient echo sequence with field of view: 230 mm^2^, matrix size: 512 × 512, slice thickness: 0.6 mm, and time of repetition (TR)/*T*_E_: 8.2 ms/3.1 ms.

Two neurosurgeons with >15 years of experience in neurosurgery and neuroradiology assessed and measured all post-surgery MRI images to quantify the final ablation area induced by LITT. On the CE MRI, the maximum cross-section of the ablation volume was selected, and the ablation diameters were determined by the two neurosurgeons who were blinded to the simulation results to verify the results (44).

### 2.3. Statistics

Paired comparisons, including those between the simulation and experimental results and between the P1-approximation and Monte Carlo simulations, were performed using paired Student’s t-tests to evaluate group differences or Pearson correlation analysis. *p* < 0.05 was considered significant.

## 3. Results

### 3.1. P1-Approximation vs. Monte Carlo simulation in modeling radiative transfer

**Figure 2:**
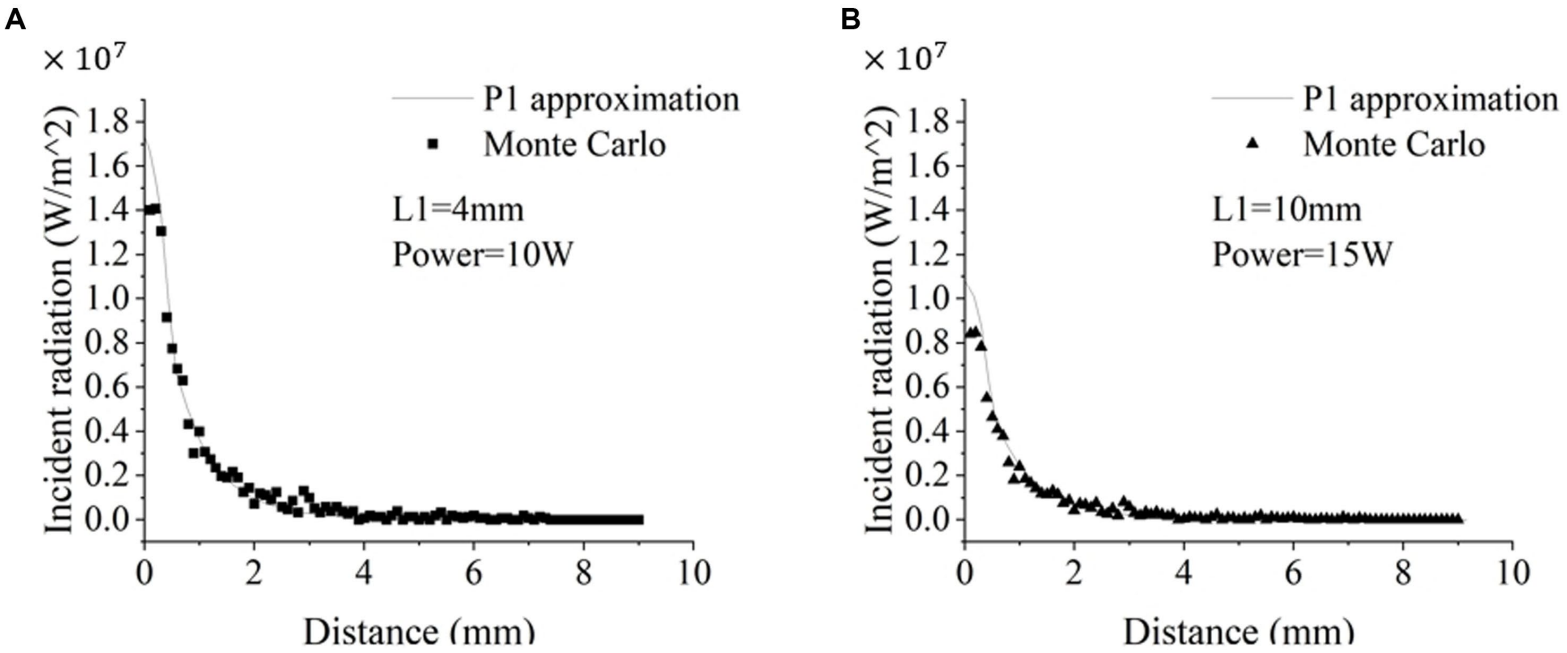
Comparison of the radiative transfer of the 1,064 nm laser calculated *via* P1-approximation and Monte Carlo simulation. Figure 1 and Table 1 illustrate the experimental setup. **(A)** The simulation parameters were a 4-mm diffusion tip with 10 W power **(A)** and a 10-mm diffusion tip with 15 W power **(B)**.

We first tested whether P1-approximation could well represent the Monte Carlo simulations of radiative transfer of 1,064 nm LITT, which can increase the computation speed in modeling radiative transfer. To compare the results from the two models, we plotted the light intensity values obtained from both models at different distances from the outer edge of the tube wall along the radial direction. P1-approximation showed great agreement with the Monte Carlo simulation at two different setups with different diffuser tip lengths (L1) and laser power (Figure 2). Further Pearson’s correlation analysis revealed a strong positive correlation between the two datasets. Specifically, the 4-mm diffuser tip with 10 W power yielded a significant and robust correlation (*r* = 0.989, *p* < 0.0001). Likewise, the 10-mm diffusion tip with 15 W power yielded a highly significant correlation (r = 0.995, *p* < 0.0001). Paired Student’s t-tests revealed no statistically significant differences for either the 4-mm diffusion tip with 10 W power (*p* = 0.6590) or the 10-mm diffusion tip with 15 W power (*p* = 0.0864). The computation time for the Monte Carlo simulation and P1-approximation for one case were approximately 805.2 ± 19.7 s (mean ± standard deviation, 10 repetitions) and 3–4 s, respectively. Thus, P1-approximation well represented the Monte Carlo simulations of radiative transfer with a 230-fold reduction in computation time. In the following sections, all simulations were performed using P1-approximation.

### 3.2. Experimental validation of temperature distribution simulation

Next, we evaluated the accuracy of the temperature distribution models by comparing them with the experimental results obtained from MRT. Because the diffusion tip length and duration and power of the laser can be adjusted in clinical practice to better cover the targeted lesion, the numerical simulations were also set to mimic the experimental settings in Table 2: two diffusion tip lengths, two laser pow We performed 180 ers, two catheter diameters, and four laser durations.

**Table 2:** Details of the 1,064 nm LITT experiments on six canine brains.

No.	Age (Months) (Sex)	Power (W)	Time (s)	Diffusion length L1 (mm)	Catheter diameter D1 (mm)
Canine 1	24 (M)	10	90	4	1.55
Canine 2	18 (M)	10	150	4	2.3
Canine 3	24 (F)	10	180	4	2.3
Canine 4	18 (F)	15	120	10	1.55
Canine 5	17 (F)	15	120	10	1.55
Canine 6	18 (M)	15	180	10	1.55

**Figure 3:**
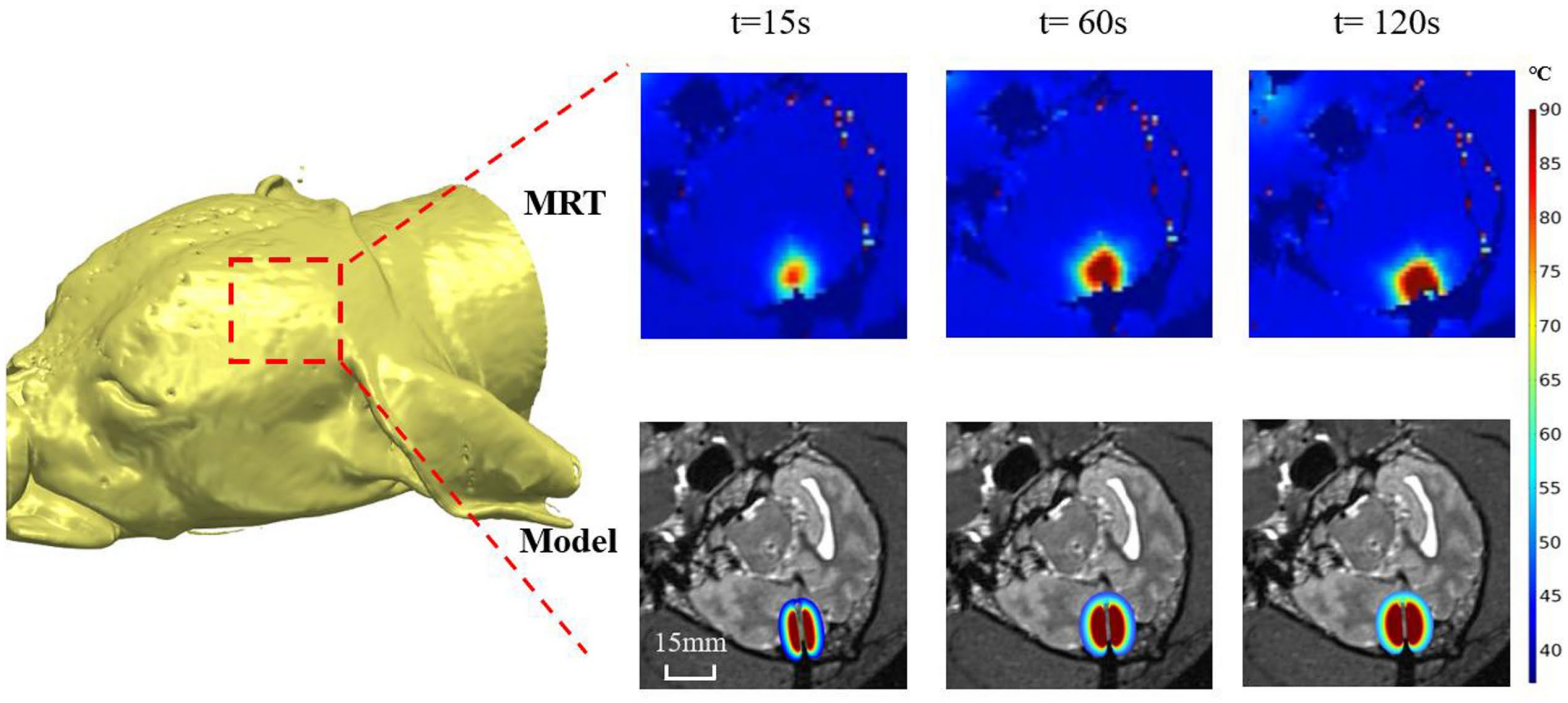
Representative example (canine 4) of the temperature distribution obtained from our proposed numerical modeling (bottom panel) and real-time MRT (upper panel) at different times (t) after the laser was turned on.

**Figure 4:**
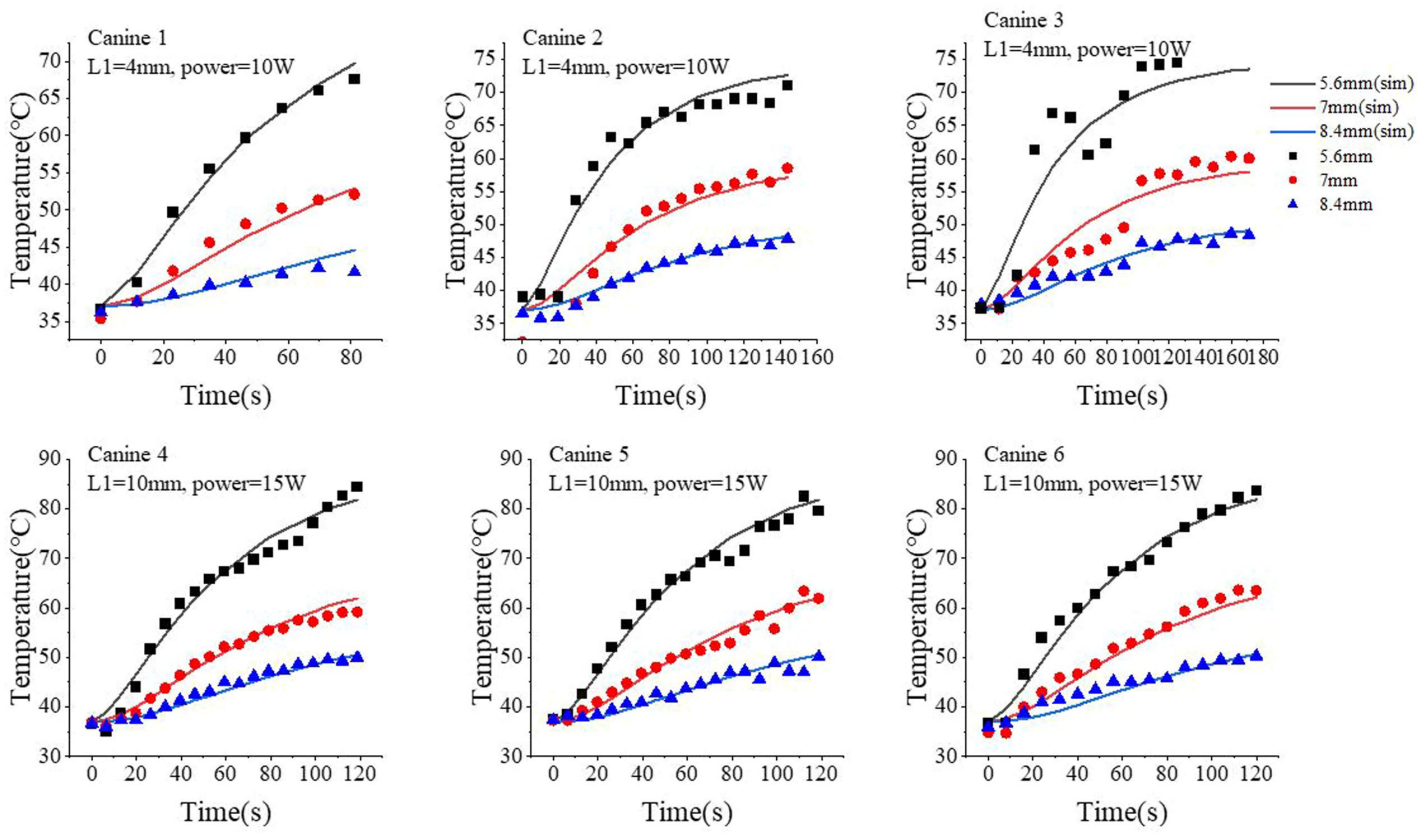
Comparison of the temperature time-course profiles obtained from our proposed simulation model and MRT measurements. Three locations were selected: 5.6 mm, 7.0 mm, and 8.4 mm from the center of optical fiber. The simulated results are shown as continuous curves; the MRT measurements are shown as scatter plots with different shapes.

Figures 3, 4 shows a representative example (canine 4) of the temperature distribution obtained from our proposed numerical modeling (bottom panel) and real-time MRT (upper panel) at different times (t) after the laser was turned on. Visual inspection showed that the temperature distributions from both methods were highly similar. The temperature at the center increased rapidly, whereas the temperature in the periphery increased gradually. As the time approached 120 s, the rate of the temperature increase slowed compared with that at 60–90 s. The MRT image also shows that when the temperature reached the outermost contour (around 55–60°C), the temperature diffusion pattern did not exhibit a relatively uniform transition diffusion, indicating the potential effect of tissue heterogeneity (see Discussion) (see Figure 3).

Next, we quantitatively compared the time-course profile of the temperature at different locations obtained from our proposed model and MRT measurements. For better visualization and generalization testing, we selected three locations at 5.6, 7.0, and 8.4 mm from the center of the fiber tip. The simulated temperature profile well approximated the measured ones *via* MRT, especially at higher temperature regions (i.e., longer time after turning on the laser). We performed 180 s of ablation during the course of the experiment on canine 6. However, a brief interruption occurred at 120 s; therefore, we analyzed only the data for up to 120 s for canine 6. At 5.6 mm, the standard deviation and median of the temperature differences between the simulated and MRT-measured temperature time-course profiles were 1.26°C and 0.19°C (canine 1), 2.79°C and 1.39°C (canine 2), 5.2°C and − 3.69°C (canine 3), 2.26°C and 0.54°C (canine 4), 1.93°C and 0.39°C (canine 5), 1.97°C and − 0.59°C (canine 6), respectively. At 7.0 mm, these values were 1.33°C and − 0.71°C (canine 1), 3.23°C and − 0.74°C (canine 2), 2.54°C and − 0.79°C (canine 3), 1.11°C and 0.42°C (canine 4), 1.45°C and − 0.26°C (canine 5), and 1.54°C and − 1.18°C (canine 6). At 8.4 mm, these values were 1.15°C and 0.66°C (canine 1), 0.68°C and 0.33°C (canine 2), 1.03°C and 0.04°C (canine 3), 0.73°C and − 0.32°C (canine 4), 1.18°C and − 0.38°C (canine 5), and 1.21°C and − 0.61°C (canine 6). At 7.0 mm, our proposed model slightly underestimated the temperature compared with that of the MRT results in the first 60 s with the laser on, but such bias was generally small (<3°C in the six canine experiments).

### 3.3. Experimental validation of the ablation region modeling

**Figure 5:**
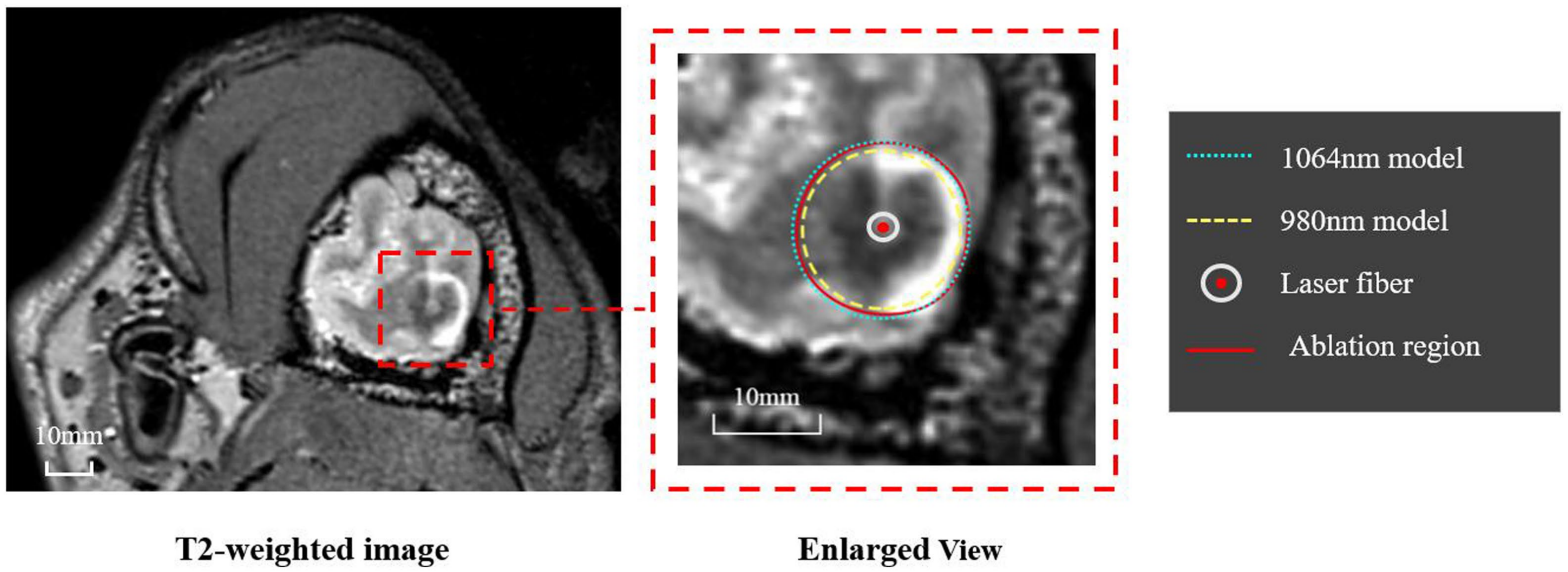
Representative example of the ablation regions obtained from T2-weighted MRI and our simulation model. The T2-weighted MRI (left panel) was used by two experienced neurosurgeons to determine the ablation region, whose boundary is shown as a red curve. The ablation regions predicted by the proposed model using 1,064 nm and 980 nm are shown as green and yellow dashed curves, respectively.

The ablation region predicted by our proposed simulation model was evaluated by comparing it with the ablation region obtained from post-LITT MRI. Here, the T2-weighted images were acquired immediately after LITT (~10 min after the laser was turned off) and used to determine the ablation region. Figure 5 shows one representative example of canine brain T2-weighted images after 1,064 nm LITT and the ablation regions drawn by the experienced neurosurgeons (red curves), predicted by our proposed model with the laser wavelength (and corresponding tissue optical parameters) set to 1,064 nm (green dashed curves). To demonstrate the sensitivity of the ablation area to laser wavelength, we performed the same simulation with a laser wavelength of 980 nm and adjusted the corresponding optical properties (Table 1), whose ablation boundary was set as a yellow dashed curve (Figure 5). Visual inspection revealed that our proposed model with the 1,064 nm laser agreed well with the T2-weighted image-measured ablation region. Expectedly, our proposed model demonstrated that the 980 nm laser had less penetration depth than did the 1,064 nm laser, which agrees with the results of previous studies (9). The results for different diffuser sizes can be discussed separately. The average percentage deviation between our model and the T2-weighted MRI measurements for the 4 mm diffuser size fiber was 0.72%, while for the 10 mm diffuser size fiber group, it was 0.03%. It can be observed that the differences between them are small. Based on the results from animal experiments, it is possible that the model can be applied to predict ablation processes with different diffuser sizes, provided accurate light distribution data are available.

**Figure 6:**
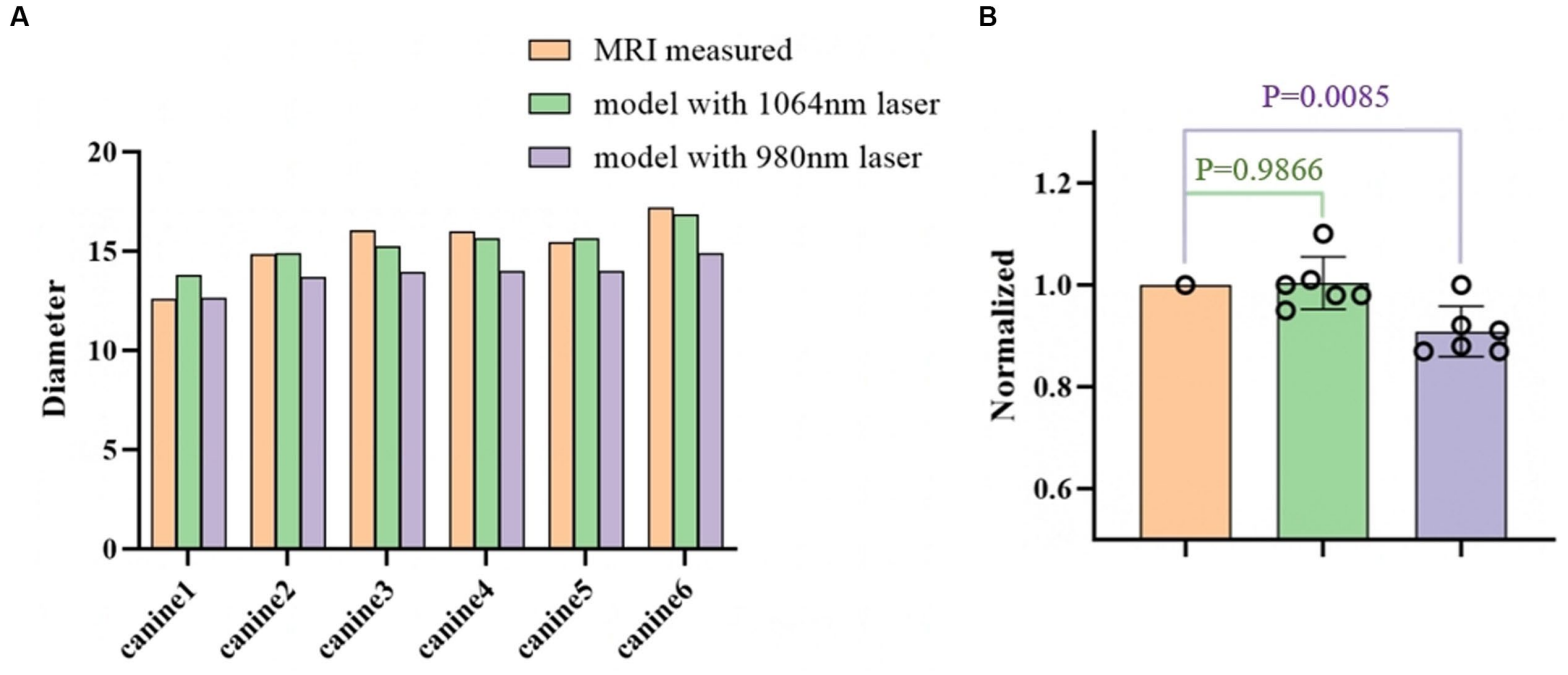
**(A)** Comparison of the ablation diameters of the 1,064 nm model, the 980 nm model, and the T2-weighted MRI measurements. **(B)** The right panel shows the normalized data based on the diameter as measured from the MR images.

Figure 6 further quantifies the diameter of the ablation area for all six canines. The proposed model with the 1,064 nm laser agreed well with the T2-weighted MRI-measured results. The differences in diameter of the ablation region between our model and the T2-weighted MRI measurements were relatively small (ranging from −1.2 mm to 0.83 mm). The predicted ablation diameter in our proposed model was further normalized by the MRI-measured ablation diameter in each canine, and the mean bias of the predicted diameter was only 0.3% (Figure 6B). Further paired Student’s t-tests showed no significant differences between the predicted and MRI-measured ablation diameters (*p* = 0.987). Additionally, in our proposed simulation model, the 980 nm laser always resulted in a smaller ablation diameter than did the 1,064 nm laser.

**Figure 7:**
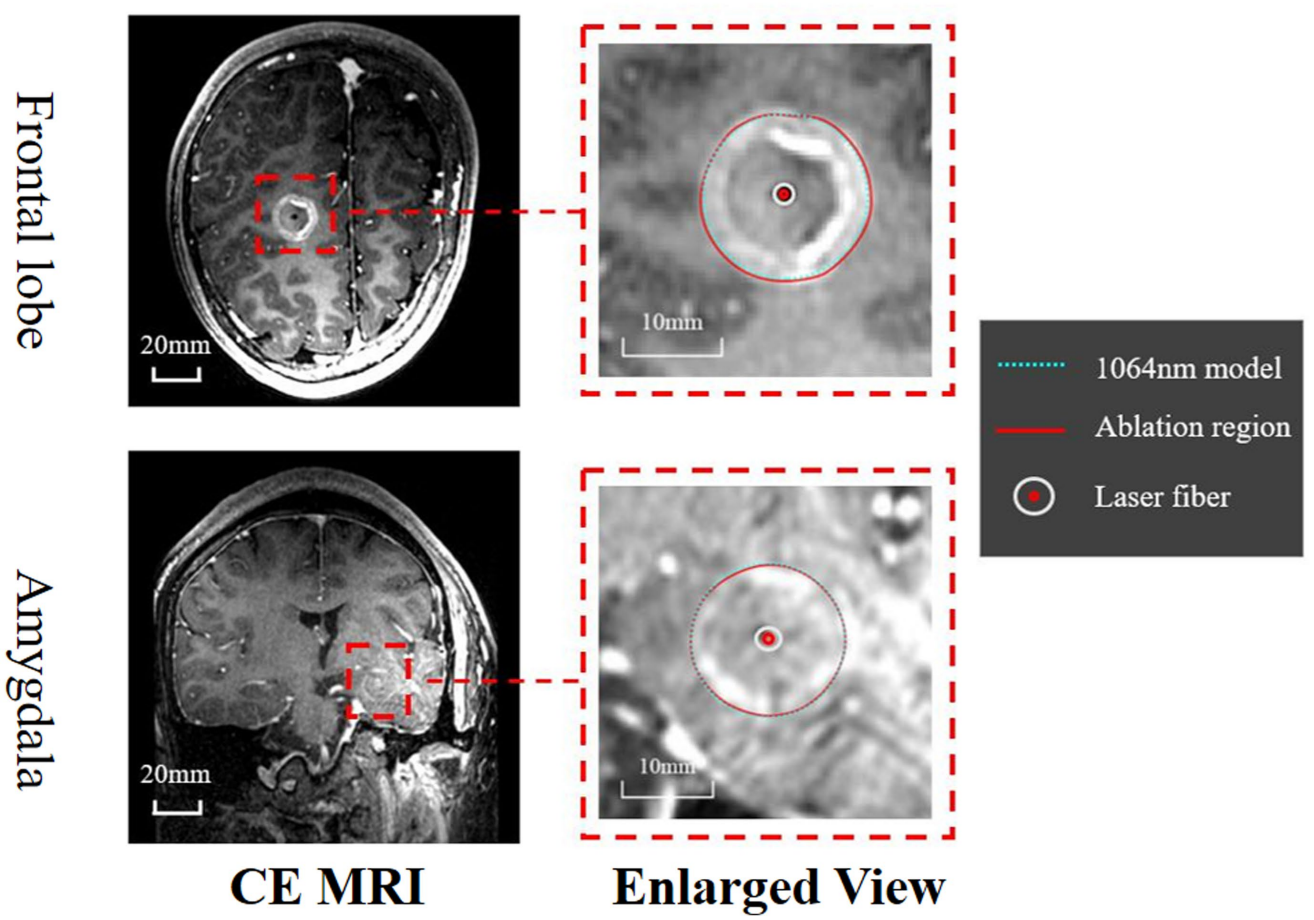
Assessment of the ablation zones was conducted across a total of six patients, with this figure illustrating the two primary ablation areas (Frontal lobe and Amygdala). The CE MRI acquired immediately after surgery was used to determine the real ablation boundary (red, curve). The ablation boundary predicted by our proposed model (1,064 nm, green dashed curve) showed good agreement with the CE MRI measurements.

**Figure 8:**
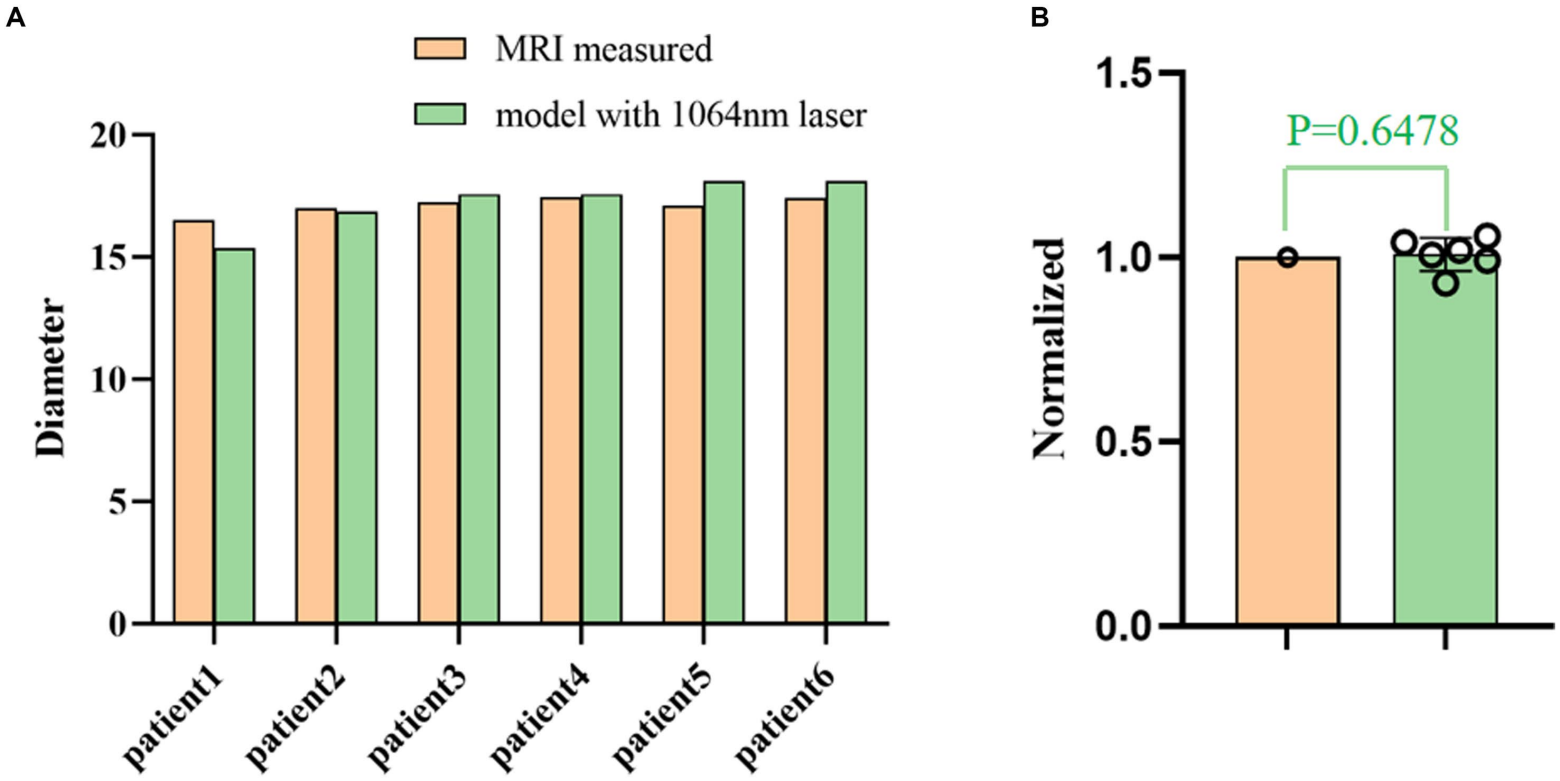
**(A)** Comparison of the ablation diameters of the 1,064 nm model and the CE MRI measurements. **(B)** The right panel shows the normalized data based on the diameter as measured from the MR images.

**Figure 9:**
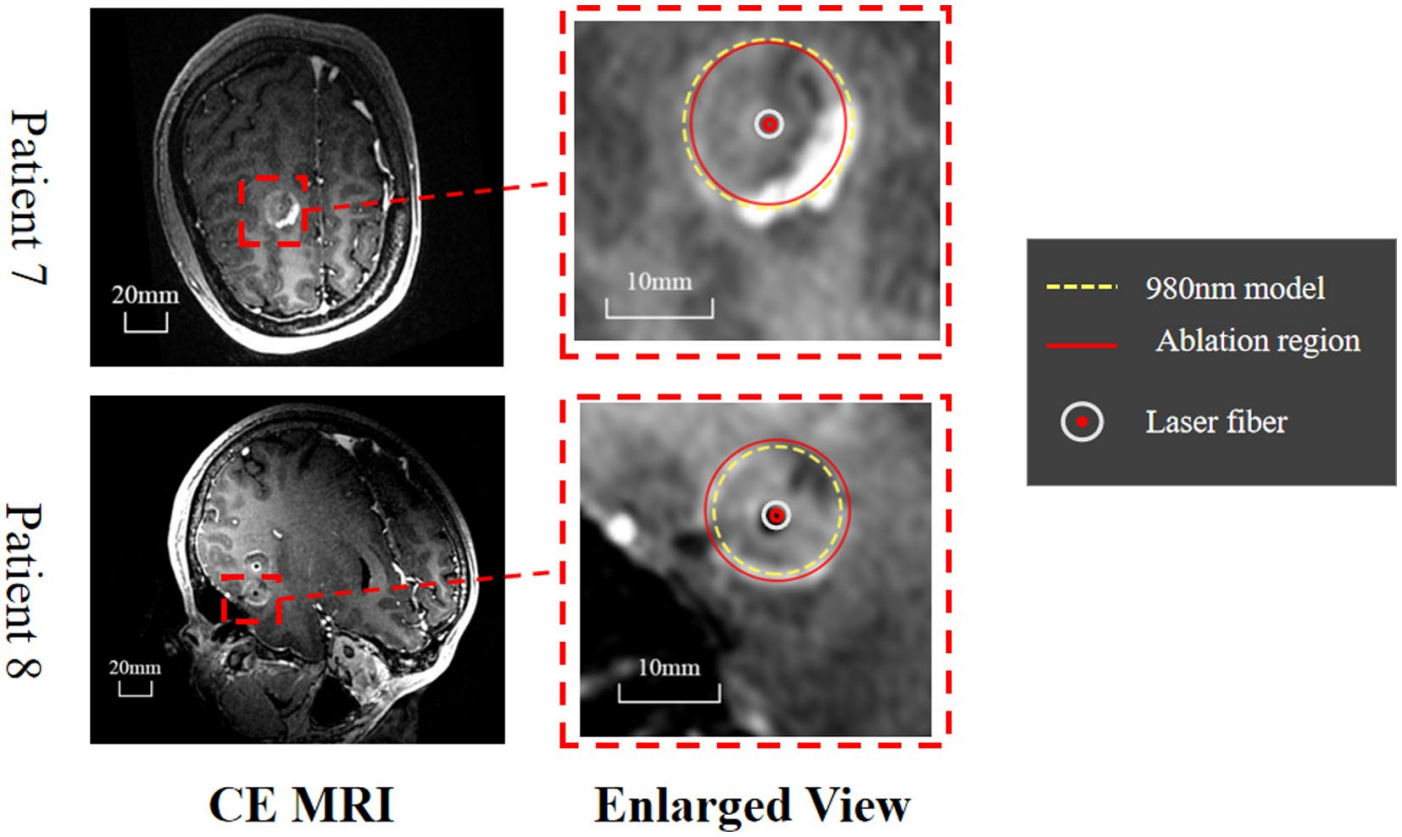
Evaluation of the predicted ablation area on two epileptic patients. The CE MRI acquired immediately after surgery was used to determine the real ablation boundary (red, curve). The ablation boundary predicted by our proposed model (980 nm, yellow dashed curve) showed good agreement with the CE MRI measurements.

Retrospective validation was performed *via* a human trial that included six epileptic patients were treated with 1,064 nm LITT (Table 3) and other two epileptic patient were treated with 980 nm LITT (Table 4). Here, the CE MRI was acquired immediately after LITT (~10 min after the laser was turned off) and used to determine the ablation region. In cases of amygdalo-hippocampal ablation, only the ablation data from the amygdala region were selected, while the data from the hippocampus region were excluded. The reason is the susceptibility of hippocampal ablation results to various external factors, including the influence of surrounding cerebrospinal fluid, ventricular structures, and other cerebral structures and vasculature. In cases, patients were treated with 1,064 nm LITT, the prediction ablation boundary showed good agreement with those drawn by the experienced neurosurgeons on the CE MRI, and the difference in diameter of the ablation area between our proposed model and the CE MRI measurements was small (ranging from −0.7 mm to 1.14 mm; Figure 7). The mean bias of the predicted diameter was only 0.81% (Figure 8A,B). Further paired Student’s t-tests showed no significant differences between the predicted and MRI-measured ablation diameters (*p* = 0.648). In other two cases, patients were treated with 980 nm LITT, the prediction ablation boundary also showed good agreement with those drawn by the experienced neurosurgeons on the CE MRI. Furthermore, the variance in ablation area diameter between our proposed model and the measurements obtained from CE MRI was minimal, with a maximum difference of 1.56 mm (Figure 9).

**Table 3:** Details of the 1,064 nm LITT experiments on six human brains.

No.	Power (W)	Time (s)	Diffusion length (mm)
Patient 1	12	240	10
Patient 2	15	180	10
Patient 3	15	240	10
Patient 4	15	240	10
Patient 5	15	300	10
Patient 6	15	300	10

**Table 4:** Details of the 980 nm LITT experiments on two human brains.

No.	Power (W)	Time (s)	Diffusion length (mm)
Patient 7	12	180	10
Patient 8	12	120	10

## 4. Discussion

LITT at 1064 nm is commonly used in practice; however, no numerical model exists that predicts the ablation region with careful *in vivo* validation. We proposed such a model with a system of finite element methods simulating heat transfer inside the brain tissue, radiative transfer from the applicator into the brain tissue, and a model for tissue damage. To quicken the computation for practical applications, we also validated P1-approximation as an efficient and fast method for calculating radiative transfer by comparing it with Monte Carlo simulation. Finally, we validated the proposed numerical model *in vivo* on six healthy canines and two human patients with epilepsy and found strong agreement between the predicted temperature profile and ablation area and the MRI-measured results. These results demonstrate that our model is feasible and reliable for predicting the ablation area of 1,064 nm LITT, which is important for presurgical planning for LITT (see Figure 9).

For radial transfer modeling, we used and validated P1-approximation, which provides faster model calculation and reduces computational time. Monte Carlo simulation with a million points and their transport through the tissue traced using random numbers and probability distributions took >13 min of computational time, whereas P1-approximation for all directions took only 3 s. This is particularly useful for large-scale models, where traditional probability distribution-based calculations can be overly time-consuming for standard computer configurations. We used a 2D axisymmetric model with a simplified geometry, which makes P1-approximation a suitable alternative to the Monte Carlo method (33). This simplification of the geometry leads to minor variations in the light propagation, but the diffusion approximation of P1 remained sufficient to predict the temperature profile and ablation extent. However, cases may occur where photon transport involves complex structures and variations, especially when reflection occurs at the interface. Monte Carlo methods can be used for these complex models such as those near the tentorium cerebellum or other solid and liquid boundaries.

The temperature increase in the 1,064 nm ablation simulation was consistent with the experimental results. This suggests that the simulation model accurately captured the physics and dynamics of the ablation process for 1,064 nm lasers. Additionally, the temperature deviation increased as the laser moved farther away from the ablation center, indicating that the accuracy of the model decreased as the distance from the center increased. However, such biases are relatively small and do not significantly affect prediction of the final ablation area, which was demonstrated in both the canine and human data. The errors in the temperature distribution predicted by the simulation may have had multiple sources. First, MRT is performed at the pixel level and represents the average temperature change over a certain range, which differs from the temperature calculation method used in the simulation. Second, errors may be caused by noise in the MR signal or changes in tissue properties during ablation, such as changes in tissue water content and tissue properties. Changes in blood perfusion can also significantly affect temperature distribution. Blood perfusion dissipates the heat generated during ablation, but changes in blood perfusion over time can alter the thermal properties of tissue, leading to inaccurate temperature predictions.

Due to the unique properties of the Proton Resonance Frequency (PRF) method, it has become the most widely used approach for monitoring water-based tissue temperatures (42, 45, 46). However, in practical MR temperature monitoring, especially in proximity to high temperatures or tissue boundaries, temperature fluctuations are prone to occur. Two key factors are of particular significance. The first factor relates to inaccuracies in core temperature measurements during the ablation process due to tissue dehydration, shrinkage, or alterations in other properties. This includes changes such as the leakage of cellular contents, thermal intracellular protein denaturation, water vaporization and tissue desiccation (25). The second factor involves the observation from previous studies that various tissues exhibit noticeable contraction or expansion behavior during thermal ablation processes (47–49). These significant tissue changes can substantially impact temperature MR measurements, leading to noticeable temperature fluctuations in high-temperature regions or tissue boundaries (50).

We also simulated the ablation region of the 980 nm laser and compared it with the results for the 1,064 nm laser. Compared with the 1,064 nm laser, the 980 nm laser ablation had a smaller but clear ablation range, partly due to the different absorption properties of laser wavelengths for water-based proteins and other tissues, resulting in an inconsistent temperature spread range and distribution pattern. The core temperature of the 980 nm laser ablation at the same power was higher, and the energy was more concentrated, resulting in sharper ablation boundaries. However, in the complex structure and high water inclusion area, based on the characteristics of 980 nm laser energy concentration, it will have better ablation form preservation and heating properties.

These results have several implications for future use of the proposed numerical model. This approach allows adjusting the laser power and duration, thereby achieving the desired ablation size while minimizing associated risks. Among the critical factors to consider, selecting the laser wavelength is particularly important. Different laser wavelengths, such as 1,064 nm and 980 nm, exhibit distinct energy absorption properties within tissues, consequently resulting in variations in their respective thermal effects. For the ablation radius, the 980 nm laser is more suitable for target areas smaller than 15 mm, where precise ablation contour boundaries are required. Conversely, for lesions with ablation diameters >15 mm and necessitating stable control over ablation boundary diffusion, the 1,064 nm laser offers notable advantages.

As for the transfer of the ablation simulation model to other human organs, it is essential to recognize that different human organs possess different anatomical structures and characteristics. Therefore, the initial step involves a clear investigation of the specific anatomical structure of the target organ. Secondly, understanding the thermal properties of the target organ, such as its thermal conductivity and heat capacity, is crucial. This includes recognizing variations in vascular distribution among different tissues and disparities in blood perfusion. Next, optical properties also exhibit significant differences among various tissues, necessitating modifications in material properties. Finally, validation through *ex vivo* experiments and *in vivo* animal studies is required to confirm the applicability of the model to different organs.

This study had several limitations, and future work on the proposed numerical model and *in vivo* validation is needed. First, changes in blood perfusion can significantly affect temperature distribution, and we assumed a constant blood perfusion in this study. Blood perfusion dissipates the heat generated during ablation, but changes in blood perfusion over time can alter the thermal properties of tissue, leading to inaccurate temperature predictions (51). Second, the homogeneous tissue model used in the simulations did not capture the heterogeneous nature of the tissue. Ablation regions invariably contain different types of tissue and structures, including blood vessels and gray/white matter junctions. It is possible to conduct separate discussions or model parameter variations for different regions within the brain ablation area, such as the hippocampus, cerebellum, frontal lobe, and cerebellar regions (46). Third, the diffusion tips assumed in the simulations were not perfectly homogeneous, especially in designs with longer or shorter diffusion tips. The non-uniformity can lead to differences in overall temperature distribution and final ablation shape in the longitudinal section (52). Fourth, tissue absorption, heat transfer rates and other tissue parameters are assumed constant throughout LITT and independent of temperature. This is inconsistent with the actual ablation process (25). After protein denaturation occurs in tissues, its absorption coefficient, scattering coefficient, and heat transfer properties change (53, 54). Fifth, since we currently lack access to instruments from other manufacturers, we are unable to facilitate comparisons between different manufacturers. However, this constitutes an important avenue for future exploration. Finally, the heat exchange with the coolant was modeled by a constant temperature wall, and the actual coolant flow rate was not considered in this study. This is only valid as long as the flow rate is sufficient to yield a small total increase in coolant temperature. In this study, the cooling capability was relatively overestimated. As coolant flows through the diffusion head, it increases by a few degrees Celsius. In the future, fluid boundaries will be used instead of isothermal boundaries to enable simulations of thermal exchange cooling and more accurately model the axial morphology. These factors should be further considered in numerical modeling and carefully validated by *in vivo* experiments (55–57).

## 5. Conclusion

Here, we presented a numerical model for predicting the ablation region of 1,064 nm LITT, addressing the lack of a comprehensive model with *in vivo* validation. The proposed model uses a system of finite element methods to model heat transfer, radiative transfer and damage within brain tissue. To improve the computational efficiency, P1-approximation was validated and applied to radiative transfer calculations, saving significant time compared with that of Monte Carlo simulations. The numerical model was validated on six healthy canines and eight human patients with epilepsy, demonstrating excellent agreement between the predicted temperature profile and ablation region and MRI measurements. Furthermore, we compared the ablation regions of 980 nm and 1,064 nm lasers. The results revealed differences in diameter range, temperature spread, and ablation boundary sharpness between the two wavelengths. These findings highlight the importance of choosing an appropriate laser wavelength based on the desired ablation size and boundary accuracy. Future research should address the limitations identified herein and further validate the model through *in vivo* experiments.

## Data availability statement

Anonymized data not published within this article will be made available by request from any qualified investigator. Requests to access the datasets should be directed to zjm135@zju.edu.cn.

## Ethics statement

The studies involving humans were approved by Human Research Ethics Committee, the Second Affiliated Hospital of Zhejiang University School of Medicine. The studies were conducted in accordance with the local legislation and institutional requirements. The participants provided their written informed consent to participate in this study.

## Author contributions

JiZ, PC, and RB conceived of the presented idea. PC, JuZ, and DS developed the theory and performed the computations. DS, ZZ, DL, and RB verified the analytical methods. All authors contributed to the article and approved the submitted version.
